# An Essay on the Mode of Treating Caries of the Teeth, to which is Added a General View of the Anatomy and Physiology of the Teeth

**Published:** 1841

**Authors:** B. A. Rodrigues

**Affiliations:** Dentist, of Charleston, S. C.


### AN ESSAY ON THE MODE

*Of treating Caries of the Teeth, to which is added a general view of the Anatomy and Physiology of the teeth.*—By B. A. Rodrigues, M. D., *Dentist, of Charleston, S. C.*

It is unfortunate for the cause alike of science and humanity, that the practice of dentistry has been left to the care of ignorance, as though the structure and diseases of the teeth were unworthy a comprehensive and philosophic investigation, and the sufferings resulting from their morbid conditions were not deserving of relief.—But we live in an age of improvement, and although the prejudices existing against the profession of the dentist, deter many from its adoption, yet intellect and industry of no common order are devoted to its prosecution; and we look forward with exulting hopes to the arrival of a period when dentistry will take its rank as a collateral branch of medical science. The sneers so commonly levelled against dentistry, find their justification in the ignorance of its professors; and such have been the miseries inflicted upon many by their want of skill and knowledge, that suffering humanity cries aloud for legislative interference. Their ready subjection to the causes of disease, and the intense pain resulting from their abnormal condition, demand for the teeth in an especial manner the protection of science; yet they have been treated with a wanton indifference, from which their importance should have sheltered them. Is it because unrestrained by legal enactments (of government) the most vulgar machinists deficient alike in intellect and cultivation, have for mere pecuniary advancement, "tendered their services to the public," that science turns with a sneer from the consideration of the teeth? And is it because accomplished quacks with "patent carbonic-dentifrices," and an endless string of puffs from persons whom nobody knows, have injured, or it may be ruined the teeth; does it follow we ask, that dentistry cannot be practiced on principle, on anatomy and physiology? So long, however, as unprofessional men, may at their pleasure assume the responsibility of the dentist, so long will injurious results follow in the train of practice. Can it be expected that men who do not understand the structure and physiology of the teeth (not to say any thing of the collateral branches) can prescribe for their diseases in a rational manner? And yet there is scarcely one of the branches of study requisite for the attainment of a degree, that is not indispensable to the dentist.

Is anatomy requisite? Can a dentist perform the simplest operation without a thorough knowledge of the structure of the teeth; the manner of their insertion, &c. &c. Is physiology requisite? How can he comprehend their morbid alterations without a proper appreciation of their healthy state? Is therapeutics necessary? Diseased states of the teeth frequently follow an impaired condition of the different functions. Can these be obviated without therapeutics? The extraction of a tooth may occasion syncope—convulsions, &c. Must not these be relieved? Is pathology necessary? The sympathies by which derangements of important functions affect the teeth, render pathology indispensable?—Chemistry? Ought not a dentist to be familiar with the chemical nature of the teeth, and the enamel, lest he prescribe an application that might destroy them? Let the legislature, then, take this matter as it should, under its own jurisdiction; let it require from the dentist knowledge. Let him undergo the regular ordeal through which the physician has to pass. If he be qualified, license him—if unqualified, do not turn loose upon the community a pretender and a quack.

### GENERAL VIEW OF THE ANATOMY AND PHYSIOLOGY OF THE TEETH.

In the human adult subject there are sixteen teeth in each maxillary bone. These are divided into four clases, viz: four *incisores*, two *cuspidati*, four *bicuspides*, and six *molares*. Varieties occur in the number of the teeth; but they are very unusual. Mr. Hunter says he once saw twenty-seven, though never more than thirty-two. The writer in Rees' Cyclopedia (article teeth) has collected several cases of an extraordinary number of teeth. Columbus, he says, has seen thirty-three. Fauchard thirty-three and thirty-four; Bourdet thirty-six; and Ingrassias thirty-six, including twenty-four grinders. Arnold met with a boy fourteen years old who had seventy-two teeth.

Each tooth is subdivided into the body or crown, the fang or root, and the neck. The body of the tooth is that portion uncovered by the gum, the root is the articulating medium, and the neck is the point of union between the body and the root. The *incisores* have one root, or fang, and the crown or body has the shape of a wedge. They are placed in the anterior part of the maxillary bone, and when viewed in connexion, form part of a circle. The *cuspidati* are the largest of the teeth, and resemble the *incisores* from which they may be distinguished by the greater pointedness of the crown.

They have been compared to a spear with lateral shoulders, but look as much like it as the clinoid processes of the sphenoid bone resemble four bed-posts, with which the fancy of the anatomist has associated them.—The *bicuspides* partake of the appearance of the *cuspidati* and the *molares*, between which they are situated.

The crown of a *bicuspid* tooth terminates in two points, and the fang is separated so as to resemble two fangs. The *molares* have four or more elevations on the crown. The molares of the lower jaw have two distinct fangs, those of the upper have three. Thomas Bell states that he has seen in a few instances, four fangs, once five fangs to the upper *molares*. The last *molar* tooth has been called the *dens sapientiæ*, because it does not appear until late in life.

In each tooth there is a cavity filled with a spongy substance, endowed with acute sensibility. The vessels and nerves which nourish this structure enter the tooth through the foramen at the extremity of the fang.

Two different substances enter into the structure of the teeth—the one *osseous*, the other called *enamel*. The bony structure is hard and dense, though its texture is the same as the rest of the osseous system. The enamel is the hardest part of the animal body, and forms only the outer covering of the crown of the teeth. The enamel is arranged in fibres closely connected. Hunter says the fibres are directed from the circumference to the centre. Haller and Majendie state that they are perpendicular to the body of the teeth; and Bichat affirms that they cannot be traced at all.

The phosphate and carbonate of lime constitute nearly the whole of the teeth in man. Of 100 parts, Majendie says 99, 5 are of earthy matter. The chemical analysis of the enamel, according to Berzelius, is as follows:

| | |
|---|---|
| Phosphate of lime, | 85.3 |
| Fluate of lime, | 3.2 |
| Carbonate of lime, | 8 |
| Phosphate of Magnesia, | 1.5 |
| Soda and muriate of soda, | 1 |
| Animal matter and water, | 1 |
| | 100 |

Of 100 parts of the bony substance, according to the same chemist, we have of

| | |
|---|---|
| Phosphate of lime, | 62 |
| Fluate of lime, | 2 |
| Carb. of lime, | 5.5 |
| Phosphate of Magnesia, | 1 |
| Soda and muriate of soda, | 1.5 |
| Gelatine and water, | 28 |
| | 100 |

The analysis of Mr. Pepys failed to detect the fluate of lime, as also the phosphate of magnesia, soda and muriate of soda, and animal matter. Mr. Brande was also unable to detect the fluate of lime, though he instituted a series of experiments for the purpose. Gay Lussac, however, asserts its existence in the enamel.

The vitality of the teeth is proved by their existence in the living animal. A recent tooth, too, taken from the socket of the jaw bone of one animal, and transplanted into that of another, will be united by a vital process. If time should elapse after extraction, and before the insertion, no union will be formed. If we judged of the vitality of the teeth, however, by their susceptibility to external influences, we would be obliged to look upon them as inorganic substances. They are endowed with a very low degree of sensibility, and in their healthy state, convey no impression to the sensorium. In the application of the file, no pain is experienced, and but for the sensation communicated to what may be called the medullary structure of the teeth, the patient would be ignorant of the operation. Grasp the tooth forcibly with the key, the patient never winces; shatter the tooth with the forceps, he does not complain. When the tip of the finger is brought in contact with a tooth, the impression is made upon the finger and not upon the tooth. If a tooth be struck with a hard body, the sound is communicated to the ear. If gently touched, the interruption to the progress of the hand is the source of sensation.—

If you strike a tooth with violence, pain is felt in its medullary structure. Cold and heat affect the pulpy substance through the bony medium. In rickets, when the jaw bone itself is distorted, the teeth are unaffected.—In mollities osseum, the teeth will be loosened by the absorption of the calcareous part of the alveolar processes, but they themselves remain sound; Bichat says, that the "diluted acids, and especially the vegetable, raise the sensibility of the enamel so much, that the least touch becomes painful a long time after their use. The teeth," he adds, "are then, as we call it, on edge." But the teeth being "on edge" is attributable to the chemical action of the acids destroying the polish upon the enamel, and thus preventing them from gliding easily over each other's surfaces.

These considerations tend strongly to show that the teeth are inorganic. Even caries is no proof of the vitality of the teeth; for may it not be referable to the chemical effect produced by the generation of morbid fluids? The pain usually attributed to the tooth, is no doubt an affection of the nerve or the medulla.

Much yet remains to be learned of the physiology of the teeth. And but for a sort of analogical certainty that every part of the animal body is vital, the phenomena of transplanting, and the effect of the removal of the nerve upon the roots of the teeth, we should be forced to infer, that the teeth were inorganic.

### OF CARIES.

Caries of the teeth may be defined to be their chemical or organic decomposition. The term caries, is manifestly incorrect; as the condition of caries of a bone, bears no resemblance to the state of the teeth, ordinarily described under that name.

This disease is not simply the death of the tooth; for when removed from the body no such appearance is observed.

Caries may attack any part of a tooth: it is most common in the crown; but it is very rarely observed in the root.

### CAUSES OF CARIES.

The most general of these, is impaired digestion, or a derangement of some of the functions connected with this important process. Dyspepsia very rapidly induces decay; and I have had frequent opportunities of observing the immediate effect produced by this disease upon the structure of the teeth. Nor are its consequences confined to a single tooth; for many become implicated, and but for the interference of art, all the bodies of the teeth would become one mass of caries.

The general exemption of the roots of the teeth from this disease, is remarkable. Animal food has been said to be a cause of caries; and in support of this it has been observed, that country negroes, who feed mostly upon vegetables, are but slightly liable to this disorder. Lord Kames, in his Sketches of Man, states "that the people of Otaheite never have a rotten tooth;" and attributes this to their living altogether upon vegetables and fish, and their abstaining from stimulating drinks and tobacco.

That uncivilized nations, living upon vegetables, are not subject to caries of the teeth, may be true; but this may be better accounted for by the temperature, than the character of the food, and the healthy condition of the functions resulting from prudence in living.

Carnivorous animals are not particularly, if at all, subject to this disease; and by fair analogy, animal food is not more apt to affect man.—The temperature of our food, however, is no doubt a fruitful source of this, as of other derangements; and the dentist may unquestionably return thanks to modern gastronomy for much of his pecuniary success in life

Want of cleanliness is a common cause of caries; and neglecting the use of the tooth-pick, frequently gives rise to this disease—small particles of food being thus allowed to remain between the teeth, the heat and moisture of the mouth occasion putrefaction, which either acts immediately upon the teeth, or engenders a condition of the gums, which indirectly affects them. The pressure of the teeth upon each other, is a very common excitant of caries; and the contact of a decayed tooth with a sound one, is very apt to produce disease of the latter, though this is staunchly denied by Bell. In enumerating the causes of caries, we must not pass over the use of the file. This instrument, in the hands of an ignorant practitioner, frequently occasions the disease under consideration. The mere contact of the teeth without pressure, is not injurious; yet it is an ordinary resort among dentists to insert the file, for the purpose of obviating this state, and thus in separating the teeth by the removal of the enamel, they nescessarily induce a diseased condition of these organs.

### TREATMENT OF CARIES.

The pain produced by a decayed tooth, as almost every one is aware from individual suffering, is of the most intense kind, and the patient in vain seeks for relief. Numerous remedies have been proposed, and quackery has not been idle in offering infallible remedies for the "tooth ache." Stimulating applications to the diseased part, are generally much resorted to; but the practice which my own experience teaches me as most beneficial, is the application of leeches to the gums, especially if there be tumefaction. A little camphor and opium, or alum and sweet spirits of nitre, will likewise frequently mitigate the sufferings. But there is no effectual remedy but the plugging of the tooth, or its removal.

In case, however, the patient will not submit to these operations, resort may be had to the means already spoken of.

The first step to be taken in the treatment of decayed teeth, should be to relieve the surrounding structures, and the teeth themselves, from all inflammation, whether idiopathic or symptomatic. To accomplish this, due attention should be paid to the state of the system. Local and general blood-letting, cathartics, &c., if necessary. After inflammatory action has been thoroughly subdued, we should proceed very carefully to remove all accumulations, whether of food or disease, from the cavity of the tooth or teeth affected.

[The instruments used for the purpose of excavating the tooth, and cutting out the decay, are blades of steel with cutting extremities fixed into ivory or pearl handles. They are of various shapes and sizes, so as to be adapted to the size of the cavity to be operated upon. There are other instruments used for this purpose, such as are termed file head borers, broaches, &c. Observations however can alone furnish the requisite information for they are innumerable, being frequently constructed for the occasion.]

When this is done, the cavity should be wiped perfectly dry with a piece of cotton attached to the point of some sharp instrument. The object then is to fill the cavity with some indestructible material. The preference is usually given to gold, because it is less liable to be corroded than any other metal used for the purpose. The mode of operating is as follows: Cut the gold leaf in small, square pieces. A piece is then taken upon the point of the filling instrument. (This is simply a piece of steel, about two inches in length, blunt at the end, and made of any shape. A number and variety of these should always be kept, so as to be adapted to different teeth and cavities.)

The gold is carefully inserted, and gently pressed so as to allow it to be well packed at the bottom of the cavity. Piece after piece is to be then introduced until the cavity is filled. Force is then to be used so as to make the plugging firm. Some Surgeon Dentists are in the habit of taking the gold on the point of the instrument, and applying immediate force, the consequence of this is injurious. The gold leaf becomes so compact and hard, that it cannot be worked and pressed so as to take on the form of the cavity; and before the tooth has been filled from the difficulty of working the gold, the operation is thought to be over, when really it is hardly more than half done. When the *lining* membrane of the tooth is exposed, the best mode of proceeding is as follows. After you have cleaned the cavity, and cut away the caries properly, apply a plate of lead over the exposed nerve, and then fill with gold. In order that this operation should prove successful, it should be performed with a great degree of care, for the smallest error will inevitably destroy the vitality of the tooth. The operation will prove unsuccessful if the least portion of decay be suffered to remain in the cavity, for this will soon act destructively on the neighbouring parts, by irritating and inflaming them. All moisture must be removed, before the introduction of the metal; for should there be any intervening fluid, galvanic effects might be the consequence, and result in irritation and inflammation of the nerve. In performing this operation, considerable tact is necessary to ensure a successful termination. Dr. Kœcker was the first who introduced lead into practice.

The following extract from his work may prove interesting. He says he "believes this metal to have a cooling and anti-inflammatory effect uppon the irritable nerve of the tooth, at least that it possesses these qualities in a greater degree than gold." "When in the commencement of my practice," he continues, "I employed gold exclusively, I was seldom successful in my labours, for inflammation, pain, &c., generally came on and obliged me in a short time to remove the tooth. I therefore resorted to the use of tin foil as an experiment, and with this metal my success was more frequent, though not what I desired it to be; for when the operation succeeded with this metal, it did not from its great liability to corrode, remain long a protection to the nerve. In all cases where tin foil is used, the tooth is preserved, but for a few years, for the saliva dissolving and uniting with the metal, may act even more destructively than the disease itself.

On recollecting the cases so generally known of leaden bullets, even when rough and battered, having remained for years imbedded in flesh, I was naturally induced to resort to the use of lead in this operation. I do not recollect to have heard that a case has been reported of any other metal remaining in the body for a long period, without exciting inflammation and suppuration in the surrounding parts. My experience has ever since strengthened the opinion I drew from these facts ; and I am more confident than ever that this substance is less irritating to living parts than any other metal."

I have had during my practice, a number of cases of exposed nerve which I treated in the above described manner, and always met with success. The following case is from my note book :

An aged gentleman, Mr. C., called on me in 1832 to have a tooth extracted which had given him pain for five or more years. He described the pain as not very severe, and as coming on at intervals. I examined the tooth* and found it much decayed on the external side, extending a little below the neck of the tooth, and consequently deprived of its gum. As the grinding surface of the tooth was in a sound state, I thought it might be made useful to him. I therefore proposed filling the tooth.—The old gentleman manifested considerable reluctance, and dwelt on the length of time the tooth had troubled him, the extent of the decay, and the difficulty as he thought of saving it. I still, however, insisted on the operation, and positively refused to extract it. He finally consented to submit to the experiment. I commenced by carefully cutting away the decayed or diseased portion of the tooth, and prepared the cavity without giving him much pain. I then applied over the exposed nerve, a plate of lead, and proceeded in the manner already mentioned. The next day he came to me, complaining that he suffered with the tooth all night, and that he was deprived of sleep.

He insisted on having it out : I succeeded once more in persuading him to suffer the tooth to remain, assuring him that the pain resulted from the cleaning of the cavity, and the expansion of the metal subsequently introduced. This satisfied him, and he took leave saying, that he would return in the morning, and if he experienced any pain during the night, he would positively have it extracted. Accordingly the next morning he came round with the full determination to have the tooth removed. I interrogated him as to the nature of the pain he experienced. He described it as dull and throbbing, and coming on at intervals. I inquired if it were as severe as before ? He answered that it was not ; but still insisted upon its extraction. I took up my instrument very reluctantly—examined the tooth—cut the gum—and applied the tooth drawers ; and when about to extract it, withdrew the instrument, and begged my patient to wait anoth-

* The second mollar of the inferior maxillæ.

er day. After a little persuasion he consented. The next day (the fourth) I saw him. He told me that the pain had been so trifling, that it was scarcely worth talking about; and from that period until the present time the tooth has never given him one moment's pain, and is as useful to him as any other. This is the most tedious case of the kind, I have met with. I might relate others, in all of which I have been successful with the treatment under consideration. I trust, however, this case will suffice to show the utility of lead, under the circumstances alluded to.

NOTE.—The Editors and Publishing Committee repeat a notice which they gave in a former number, that they do not hold themselves responsible for the sentiments expressed in the articles of this Journal not written by themselves. They often admit articles, the correctness of which they altogether doubt, for the sake of free discussion.—PUB. COM.

